# Adjuvant COX inhibition augments STING signaling and cytolytic T cell infiltration in irradiated 4T1 tumors

**DOI:** 10.1172/jci.insight.165356

**Published:** 2024-05-21

**Authors:** Lisa A. Ridnour, Robert Y.S. Cheng, Noemi Kedei, Veena Somasundaram, Dibyangana D. Bhattacharyya, Debashree Basudhar, Adelaide L. Wink, Abigail J. Walke, Caleb Kim, William F. Heinz, Elijah F. Edmondson, Donna O. Butcher, Andrew C. Warner, Tiffany H. Dorsey, Milind Pore, Robert J. Kinders, Stanley Lipkowitz, Richard J. Bryant, Jens Rittscher, Stephen T.C. Wong, Stephen M. Hewitt, Jenny C. Chang, Aliaa Shalaby, Grace M. Callagy, Sharon A. Glynn, Stefan Ambs, Stephen K. Anderson, Daniel W. McVicar, Stephen J. Lockett, David A. Wink

**Affiliations:** 1 Cancer Innovation Laboratory, CCR, NCI, NIH, Frederick, Maryland, USA.; 2 Collaborative Protein Technology Resource (CPTR) Nanoscale Protein Analysis, OSTR, CCR, NCI, NIH, Bethesda, Maryland, USA.; 3 Optical Microscopy and Analysis Laboratory, Cancer Research Technology Program, Frederick National Laboratory for Cancer Research, and; 4 Molecular Histopathology Laboratories, Leidos Biomedical Research Inc. for the National Cancer Institute, Frederick, Maryland, USA.; 5 Laboratory of Human Carcinogenesis, CCR, NCI, NIH, Bethesda, Maryland, USA.; 6 Imaging Mass Cytometry Frederick National Laboratory for Cancer Research, and; 7 Office of the Director, Division of Cancer Treatment and Diagnosis, National Cancer Institute, Frederick, Maryland, USA.; 8 Women’s Malignancy Branch CCR, NCI, NIH, Bethesda, Maryland, USA.; 9 Department of Urology, Nuffield Department of Surgical Sciences, University of Oxford, Oxford, United Kingdom.; 10 Institute of Biomedical Engineering, Big Data Institute, Ludwig Oxford Branch, University of Oxford, Oxford, United Kingdom.; 11 Houston Methodist Neal Cancer Center, Weill Cornell Medical College, Houston Methodist Hospital, Houston, Texas, USA.; 12 Laboratory of Pathology CCR, NCI, NIH, Bethesda, Maryland, USA.; 13 Discipline of Pathology, Lambe Institute for Translational Research, School of Medicine, University of Galway, Galway, Ireland.; 14 Basic Science Program, Frederick National Laboratory for Cancer Research, Frederick, Maryland, USA.

**Keywords:** Inflammation, Oncology, Adaptive immunity, Breast cancer

## Abstract

Immune therapy is the new frontier of cancer treatment. Therapeutic radiation is a known inducer of immune response and can be limited by immunosuppressive mediators including cyclooxygenase-2 (COX2) that is highly expressed in aggressive triple negative breast cancer (TNBC). A clinical cohort of TNBC tumors revealed poor radiation therapeutic efficacy in tumors expressing high COX2. Herein, we show that radiation combined with adjuvant NSAID (indomethacin) treatment provides a powerful combination to reduce both primary tumor growth and lung metastasis in aggressive 4T1 TNBC tumors, which occurs in part through increased antitumor immune response. Spatial immunological changes including augmented lymphoid infiltration into the tumor epithelium and locally increased cGAS/STING1 and type I IFN gene expression were observed in radiation-indomethacin–treated 4T1 tumors. Thus, radiation and adjuvant NSAID treatment shifts “immune desert phenotypes” toward antitumor M1/TH1 immune mediators in these immunologically challenging tumors. Importantly, radiation-indomethacin combination treatment improved local control of the primary lesion, reduced metastatic burden, and increased median survival when compared with radiation treatment alone. These results show that clinically available NSAIDs can improve radiation therapeutic efficacy through increased antitumor immune response and augmented local generation of cGAS/STING1 and type I IFNs.

## Abstract 2

Inexpensive, clinically available NSAIDs may provide alternative and effective immunetherapeutic options for the treatment of aggressive, chemoresistant tumors.

## Introduction

The immune checkpoints programmed cell death 1/programmed death ligand 1 (PD-1/PD-L1) are key regulators of the immune system that are crucial for self-tolerance and are exploited by tumors for their survival and disease progression. In recent years, checkpoint inhibitors have been employed in cancer therapies to limit immunosuppression and promote antitumor M1/Th1 immune responses; this has improved treatment efficacy and clinical outcomes of some tumors (1). However, there remain a substantial fraction of cancers that do not respond to immunotherapeutic intervention, such as triple-negative breast cancer (TNBC). Recent reports have shown that patients with TNBC present with elevated PD-L1 tumor expression; however, only 8%–20% respond to checkpoint inhibitor therapy (2, 3). These observations suggest that other mechanisms prevent maximum tumor immune response.

The efficacies of conventional chemotherapies and focused irradiation can be enhanced by a proactive immune response. For example, augmented radiation-induced tumor growth delay by TGF-β neutralizing antibody is completely abated by CD8^+^ or CD4^+^ T cell depletion, implicating the requirement of cytolytic T cells for improved radiation therapeutic efficacy (4). Similarly, inhibition of the immunosuppressive enzyme indoleamine 2,3-dioxygenase (IDO) also improves standard of care therapy (5). This observation is supported by recent studies in melanoma demonstrating that elevated products of tryptophan catabolism by IDO limits responsiveness of immune-based therapies (6). In addition, targeting IL-10 increased the therapeutic efficacies of radiation and CpG treatments (7, 8). Importantly, IL-10 blockade increased the survival of tumor-bearing mice by 30% (9). These findings demonstrate roles of alternative immune-suppressive pathways in addition to PD-1/PD-L1 that, when targeted, enhance proinflammatory immune responses and therapeutic efficacies.

In addition to the immunosuppressive pathways described above, the inducible forms of nitric oxide synthase (iNOS or NOS2) and cyclooxygenase-2 (COX2) modulate inflammatory microenvironments (10, 11). Interestingly, elevated tumor expression of NOS2 and COX2 predicts poor survival in estrogen receptor–negative (ER^–^) patients (12–14). Both NOS2 and COX2 promote drug resistance, metastasis, angiogenesis, and an immunosuppressive tumor microenvironment (TME) (11, 14–17). Moreover, NOS2 and COX2 fortify their expression in a feed-forward manner as they drive multiple oncogenic pathways, including Erk, Akt, HIF1α, NF-κB, and TGF-β (SMAD), through generation of different cytokines including TNF-α, GM-CSF/G-CSF, IL-6, and IL-8 (11, 17–20). TCGA pathway analysis of NOS2/COX2^+^ tumors implicates many immune pathways, including IL-17A, IFN-γ, IL-1β, and TLR signaling in ER^–^ breast cancer (19). These data indicate that NOS2^+^ and COX2^+^ cancers induce an active immune response involving Th1- and Th17-related pathways. Given that inflammation is commonly encountered in breast cancers, the poor prognosis predicted by increased tumor expression of NOS2 and/or COX2 may in part be due to an altered tumor immune microenvironment that limits treatment efficacy and clinical outcome. Neoadjuvant therapy is a standard of care with chemo and/or radiation treatments, and this suggests that NOS2/COX2 inhibition could provide a new opportunity for improved therapeutic efficacies. Toward this end, a recent phase 1/2 clinical trial showed an improved clinical outcome defined by an overall response rate of 45.8% in patients with drug-resistant, locally advanced breast cancer (LABC) and metaplastic TNBC who received the NOS inhibitor L-NMMA and low-dose aspirin combined with taxane (21). Importantly, 27.3% of treated patients with LABC achieved pathological complete response at surgery where remodeling of the tumor immune microenvironment was observed in therapeutic responders (21).

In addition to NOS inhibitors, nonsteroidal antiinflammatory drugs (NSAIDs) have been shown to delay tumor growth, limit chemoresistance, and reduce metastasis (22). When administered in conjunction with irradiated (120 Gy) 4T1 cells, indomethacin (INDO) dramatically induced a potent antitumor immune response (23). Antitumor immunity was long lasting in 4T1 tumor–bearing mice, where 48% of mice were resistant to a second 4T1 challenge. Remarkably, this vaccine response was effective against high but not low COX2-expressing tumor cells. Since COX2 is highly expressed in many tumors, including TNBC, these results suggest that targeting COX2 may be clinically beneficial (23).

Herein, we explored the effects of pan-NOS and -COX inhibitors on radiation therapeutic efficacy in the murine 4T1 TNBC tumor model. When administered after irradiation (IR), the pan-NOS inhibitor L-NAME modestly enhanced radiation-induced tumor growth delay but did not affect lung metastatic burden. In contrast, COX inhibition by INDO reduced primary tumor growth and decreased metastasis as a single agent and in combination with focused tumor irradiation. Using CODEX imaging, elevated densities of infiltrating CD8^+^ and CD4^+^ T cells were observed in INDO- and 6 Gy + INDO combination–treated tumors, and these elevated densities were sustained through 23 days after IR. In addition, infiltration patterns differed between treated and control tumors where high leukocyte populations defined fully inflamed tumors in response to treatment. In contrast, untreated control tumors exhibited immune deserts characterized by low levels of leukocyte infiltrates. Examination of leukocyte infiltrates at earlier time points revealed elevated leukocyte infiltration at day 7 after IR. Importantly, these results are supportive of elevated CD8^+^ T cell density and penetration into tumor parenchyma observed in COX2_lo_ TNBC tumors. Three factors that appear to be important include augmented cGAS/STING and type I IFN, as well as increased tumor infiltration of cytolytic CD8^+^ T cells in combination-treated tumors. Together, these results suggest that improved antitumor immune response can be achieved by adjuvant COX2 inhibition in conjunction with radiation, which may provide a beneficial therapeutic option for treatment of TNBC and other high COX2–expressing tumors.

## Results

### Elevated tumor COX2 expression limits radiation therapeutic efficacy and leukocyte infiltration in TNBC tumors.

**Figure 1:**
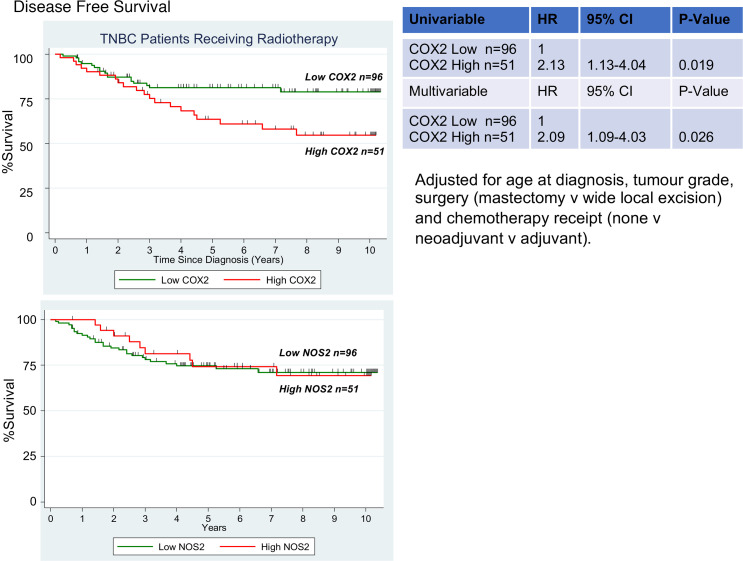
Association between tumor COX2 expression and breast cancer survival. Kaplan-Meier and log rank test were used to determine cumulative disease-free survival curve of patients with TNBC (*n* = 147) by COX2 status; when compared with low COX2 tumor expression (*n* = 96), high COX2 (*n* = 51) predicted poor survival among patients who had received fractionated radiation doses totaling 50 Gy. *P* = 0.026. Elevated NOS2 tumor expression had no predictive value in the same patients.

More than 60% of patients with cancer receive radiation therapy as part of their treatment regimen (24). In TNBC, radiation therapy is considered an option in locally advanced cases (25). Given that elevated NOS2/COX2 tumor expression is a strong predictor of poor survival among ER^–^ patients and patients with TNBC, we postulated that elevated expression of these enzymes might limit clinical outcome in conjunction with radiation treatment. This hypothesis was explored in a cohort of patients with TNBC (*n* = 147) previously treated with fractionated radiation doses totaling 50 Gy (26). When stratifying for COX2 expression, Kaplan-Meier survival analysis demonstrated that elevated COX2 tumor expression predicted poor survival (HR = 2.09; *P* = 0.026) (Figure 1). In contrast, elevated tumor NOS2 expression had no predictive value in the same patients (Figure 1). These results suggest that pharmacological COX2 inhibition could improve radiation therapeutic efficacy in patients with TNBC with high COX2–expressing tumors.

**Figure 2:**
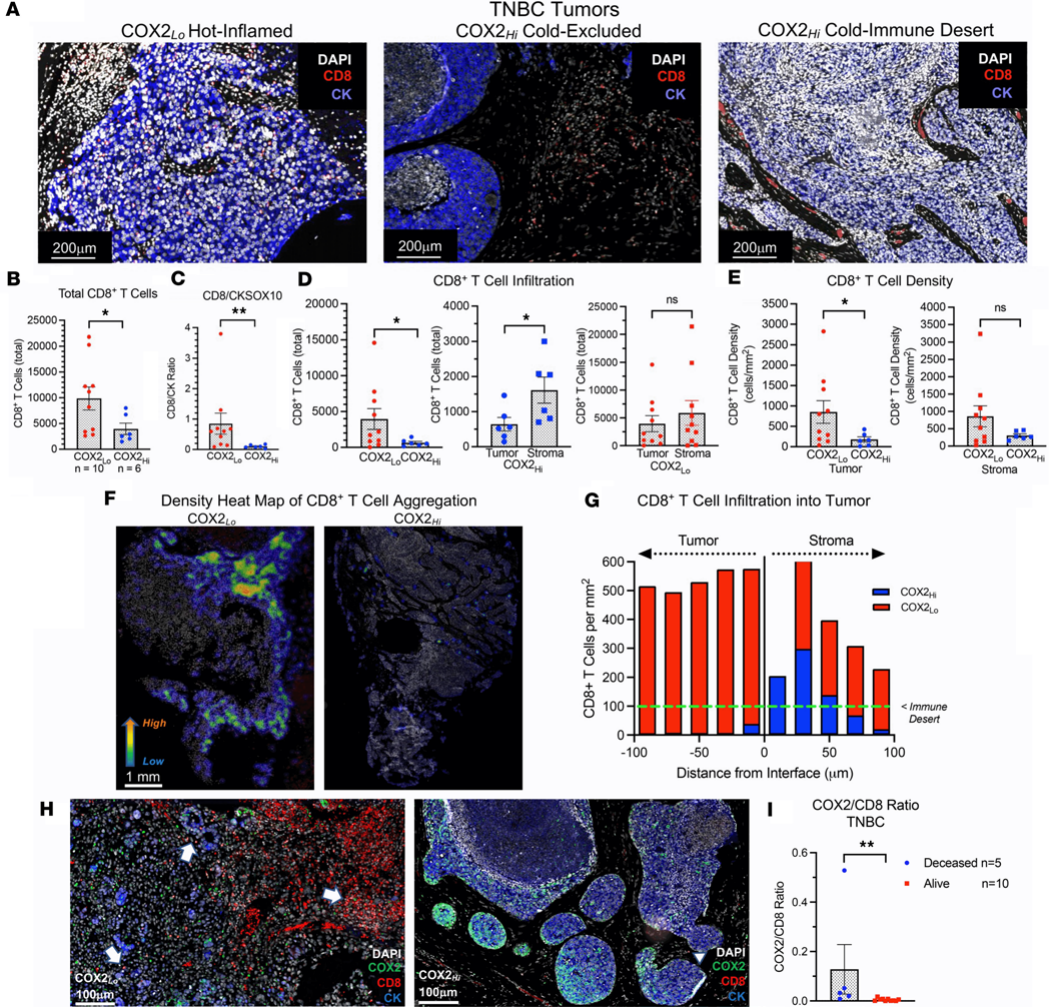
CD8^+^ T cell spatial distribution in COX2_hi_ and COX2_lo_ expressing tumors. (**A**) CD8^+^ T cells (red stain), CK tumor marker (blue stain), and DAPI (white stain) showing COX2_lo_ “Hot-Inflamed” tumor with high CD8^+^ T cell penetration into tumor epithelium, COX2_hi_ “Cold-Excluded” tumor showing CD8^+^ T cells restricted to stroma, and COX2_hi_ “Cold-Immune Desert” showing few or absence of CD8^+^ T cells in the tumor epithelium. Scale bars: 200 μm. (**B**) CD8^+^ T cell quantification showing increased total CD8^+^ T cells in COX2_lo_ (red circles *n* = 10) versus COX2_hi_ (blue circles *n* = 6) tumors. (**C**) Increased CD8^+^ T cell/tumor CKSOX10 ratio in COX2_lo_ (red) versus COX2_hi_ (blue) tumors. (**D**) The left graph shows significantly elevated CD8^+^T cell infiltration in COX2_lo_ versus COX2_hi_ tumors. The middle graph shows significantly reduced CD8^+^ T cell infiltration in COX2_hi_ annotated tumor regions where CD8^+^ T cells are highly stroma restricted. The right graph shows no significant difference between CD8^+^ T cells in tumor versus stroma regions in COX2_lo_ tumors. (**E**) CD8^+^ T cell density per mm^2^ localized in tumor- (left) or stroma-annotated (right) regions in COX2_lo_ (red) versus COX2_hi_ (blue) tumors. (**F**) Density heat map showing elevated CD8^+^ T cell aggregation in COX2_lo_ versus COX2_hi_ tumors. Scale bar: 1 mm. (**G**) Increased number of CD8^+^ T cells infiltrating from tumor-stroma interface into tumor epithelium in COX2_lo_ (red bar) versus COX2_hi_ (blue bar) tumors. (**H**) COX2_lo_ expressing tumors (left panel) exhibit dramatically increased number and penetration of CD8^+^ T cells into tumor epithelium (white arrows). In contrast, CD8^+^ T cell (white arrowhead) in COX2hi tumors (right panel) are stroma restricted. DAPI (white), COX2 (green), CD8^+^ T cell (red), and CKSOX10 tumor marker (blue) are shown. (**I**) Increased COX2/CD8^+^ T cell ratios in patients with TNBC who succumbed to disease versus those who survived (Deceased versus Alive) at 5 years after diagnosis. *P ≤ 0.05, **P ≤ 0.0075 using Mann-Whitney *U* or Welch’s test.

Tumor leukocyte infiltration and spatial localization predicts clinical outcomes (2, 27). However, elevated tumor COX2 is known to promote an immunosuppressive TME (11, 16, 28). To further explore the role of COX2 in altered tumor immune responses, we used InSituPlex multiplex imaging to examine the density, infiltration, and localization of CD8^+^ T cells in COX2_hi_ versus COX2_lo_ TNBC tumors (13, 14). The expression levels and spatial localization of immune biomarkers including CD3, CD4, CD8, CD68, FOXP3, PD-1, and PD-L1 (Supplemental Figure 1; supplemental material available online with this article; https://doi.org/10.1172/jci.insight.165356DS1) were examined relative to the tumor marker CK/SOX10 in 16 TNBC COX2_hi_ versus COX2_lo_ expressing tumors. Using this approach, Figure 2A shows 3 types of tumor immune microenvironments including (a) increased CD8^+^ T cell penetration into the tumor core in COX2_lo_ tumors (Hot-Inflamed), (b) CD8^+^ T cells that were spatially restricted to tumor stroma in COX2_hi_ tumors (Cold-Excluded), and (c) the sparse distribution or absence of CD8^+^ T cells in the tumor epithelium in COX2_hi_ tumors (Cold-Immune Desert). To further explore correlations between COX2 and CD8^+^ T cell density, total CD8^+^ T cells and CD8-to-CK/SOX10 (CK tumor marker) ratios were quantified in COX2_lo_ (red circles) versus COX2_hi_ (blue circles) tumors. This quantitative approach revealed approximately 2- to 4-fold increases in total CD8^+^ T cell number (Figure 2B) and CD8/CK ratio (Figure 2C) respectively, in COX2_lo_ tumors when compared with COX2_hi_ tumors. The spatial distribution of CD8^+^ T cells influences clinical outcome (2). Next, we examined CD8^+^ T cell spatial distribution and found elevated CD8^+^ T cell penetration into COX2_lo_ tumor epithelium when compared with COX2_hi_ tumor epithelium (Figure 2D, left). Moreover, CD8^+^ T cells restricted to COX2_hi_ tumor stroma (blue squares) were increased when compared with CD8^+^ T cells that infiltrated COX2_hi_ tumor epithelium (blue circles) (Figure 2D, middle). However, the number of CD8^+^ T cells in COX2_lo_ tumor epithelium (red circles) was not significantly different than those in COX2_lo_ tumor stroma (red squares) (Figure 2D, right). In addition, CD8^+^ T cell density in COX2_lo_ tumor epithelium (850 cells/mm^2^) was higher than COX2_hi_ tumor epithelium (189 cells/mm^2^) (Figure 2E). These results suggest that increased COX2 tumor expression promotes increased areas of immune deserts (100 cells/mm^2^) as previously described (2). Moreover, density heatmaps show mixed landscapes of stroma and marginally restricted CD8^+^ T cell aggregates as well as immune desert regions near or below 100 cells/mm^2^ in COX2_hi_ tumors (Figure 2, F and G). In contrast, COX2_lo_ tumors showed increased CD8^+^ T cell penetration (Figure 2F) and aggregation approaching 600/mm^2^ when quantified at depths of 100 μm beyond the tumor-stroma interface (Figure 2G). Further examination of the COX2/CD8 spatial relationship in COX2_hi_ tumors demonstrated elevated COX2 expression bordering the tumor margin that appeared to restrict CD8^+^ T cells penetration into the core (Figure 2H). These observations are consistent with elevated CD8^+^ T cells restricted to COX2_hi_ tumor stroma when compared with those that infiltrated into COX2_hi_ tumor epithelium (Figure 2D). In contrast, dramatically increased CD8^+^ T cell penetration into the tumor epithelium of COX2_lo_ tumors was observed (Figure 2H). Importantly, quantified COX2/CD8 ratios were increased in deceased patients with TNBC when compared with those who survived at 5 years after diagnosis (Figure 2I). In addition to CD8^+^ T cells, CD4^+^ T cells were also increased in COX2_lo_ tumors (Supplemental Figure 2). Together, these results show that elevated COX2 expression correlates with limited CD8^+^ T cell density and infiltration into the tumor epithelium in TNBC and that this spatial orientation is a limiting factor in clinical outcome.

### COX inhibition improves radiation therapeutic efficacy and leukocyte infiltration in 4T1 tumor–bearing mice.

**Figure 3:**
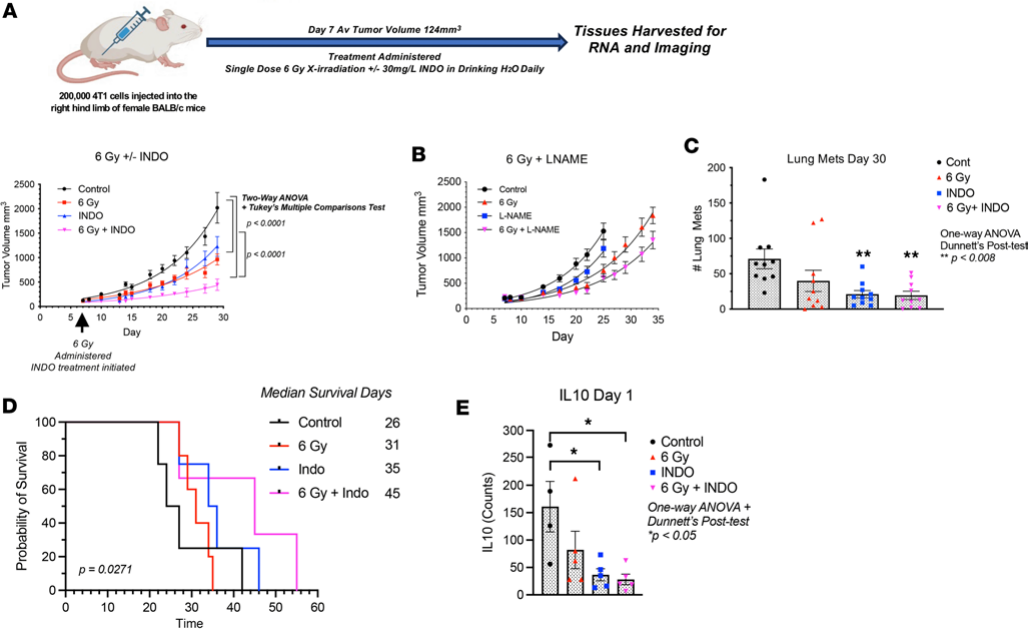
Antitumor effect of 6 Gy radiation ± INDO in 4T1 tumor–bearing mice. (**A**) A single dose of 6 Gy irradiation as well as daily INDO treatments were given on day 7 following tumor injection. After tumor irradiation, the mice were returned to their cage and given INDO (30 mg/L) in the drinking water, which continued for the duration of the experiment. The tumor growth curve shows intermediate growth delays associated with single agent 6 Gy and INDO treatments while 6 Gy + INDO combination treatment abated tumor growth. Two-way ANOVA with Tukey’s multiple-comparison test was used to determine significant changes in tumor growth. (**B**) Modest enhancement of 6 Gy–induced growth delay by the pan-NOS inhibitor L-NAME. (**C**) INDO alone and 6 Gy + INDO treatments reduce lung metastatic burden when compared with control untreated mice. One-way ANOVA with Dunnett’s post hoc test was used was used. (**D**) Improved median survival associated with 6 Gy + INDO combination treatment. *P* = 0.0271 log rank test for trend. (**E**) RNA-Seq gene expression showing reduced IL-10 gene expression in INDO and 6 Gy + INDO–treated tumors. One-way ANOVA with Dunnett’s post hoc test were used *P < 0.05.

Given that elevated COX2 tumor expression limited radiation therapeutic efficacy (Figure 1) and that reduced CD8^+^ T cell infiltration and increased COX2/CD8 ratios were observed in COX2_hi_ tumors and deceased patients (Figure 2), COX2 effects on radiation therapeutic efficacy was further examined in 4T1 tumor–bearing mice. The murine 4T1 model was used because the disease progression of 4T1 tumors closely follows TNBC disease progression in humans, as defined by spontaneous metastasis from the primary tumor to lymph nodes, blood, liver, lung, brain, and bone (29). 4T1 tumor–bearing mice were treated with 1 dose of 6 Gy x-rays (Supplemental Figure 3), and this effectively induced a tumor growth delay in 4T1 tumor–bearing mice that could be further augmented by combination treatment. A single dose of 6 Gy x-rays gave the same response as 30 Gy total dose administered in 6 Gy dose fractions in 4T1 tumor–bearing mice as reported by Vanpouille-Box et al. (4). Given that the dose enhancement ratio (DER; the slope of tumor growth in treated/control; Supplemental Table 1) in our study was consistent with that of Vanpouille-Box (4), along with limited stress to the mice and the radiation sensitivity of T cells (30), we used the single-dose method. INDO — a potent, clinically available NSAID — was used for combination treatment based upon (a) its accumulation in high COX2–expressing tumors due to its slow rate of release from the COX2 enzyme and (b) its ability to increase expression of the PGE2 consumptive enzyme PGDH (31, 32). When compared with control untreated mice, intermediate tumor growth delays were observed in mice treated with 6 Gy or INDO as single agents (Figure 3A). However, when administered in combination, 6 Gy + INDO abated tumor growth through 30 days as shown in Figure 3A. Similar combination effects were observed in EMT-6 (BALB/c) and EO771 (C57BL/6) tumor–bearing mice (Supplemental Figure 4). Our earlier work reported DER in a nonmetastatic squamous cell carcinoma (SCC) murine model that showed improved radiation-induced tumor growth delay by the pan-NOS inhibitor L-NAME (DER 1.8), which involved abated IL-10 expression and increased CD8^+^ T cell number and activation (7). Herein, the NOS inhibitor L-NAME modestly enhanced the radiation-induced growth delay (DER 1.4) (Figure 3B) with no effect on metastatic burden (data not shown). In contrast, when compared with untreated controls, single-agent INDO and 6 Gy + INDO combination treatment reduced lung metastatic burden (Figure 3C) and improved median survival (Figure 3D) in 4T1 tumor–bearing mice. In addition, RNA-Seq gene expression analysis showed significantly reduced IL-10 gene expression by INDO treatment when compared with control untreated mice (Figure 3E), and this supports earlier studies and further implicates COX2 in the regulation of immune suppression and adaptive immunity (7, 9). Importantly, these results support the Kaplan-Meier analysis shown in Figure 1 demonstrating improved radiation therapeutic efficacy in patients with TNBC with low COX2–expressing tumors.

**Figure 4:**
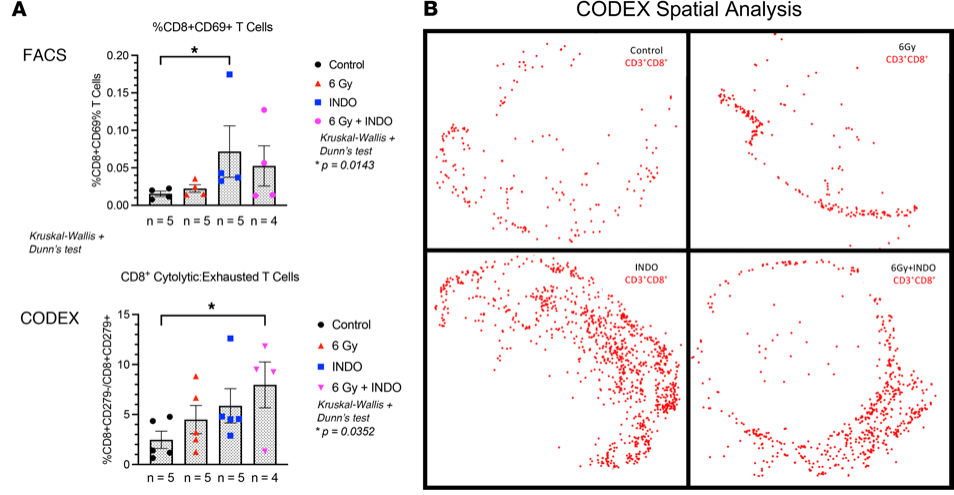
FACS and CODEX analyses show increased tumor infiltrating CD8^+^ T cells on day 7 after IR. (**A**) FACS and CODEX analysis show increased CD8^+^CD69^+^ (active) T cells and increased cytolytic/exhausted CD8^+^ T cell ratios, respectively. (**B**) CD8^+^ T cell spatial distribution, where red dots represent the detection of > 1 CD8^+^ cell marker in a 25 μm diameter circle and the spatial location of the migrating cells. *P < 0.05 using Kruskal-Wallis with Dunn’s post hoc test.

**Figure 5:**
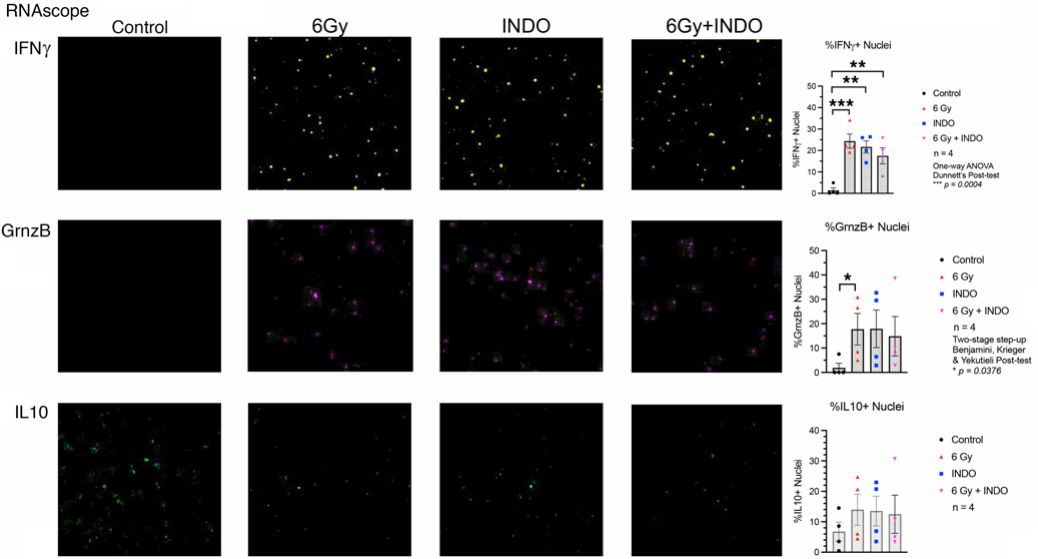
RNAScope analysis of IFN-γ, GrnzB, and IL-10. Increased expression of IFN-γ and GrnzB supportive of elevated cytolytic CD8^+^ T cell phenotypes were measured in treated tumors on day 7 after IR. No significant changes in IL-10 expression were observed. *P < 0.05, **P < 0.006, ***P = 0.0004 using 1-way ANOVA with Dunnett’s post hoc test or Kruskal-Wallis with Benjamini, Krieger, & Yekutieli 2-stage step-up post hoc test. Magnification, ×20.

Since reduced IL-10 levels improved adaptive immunity (7, 9, 33), we further explored the effect of COX2 blockade on adaptive immune response in the 4T1 TNBC murine model. The expression, density, and spatial location of lymphocyte biomarkers were evaluated using RNA-Seq, FACS, and Multiplex CODEX imaging in control and 6 Gy ± INDO–treated tumors harvested 7 days after IR. When compared with untreated controls, FACS analysis showed elevated CD8^+^CD69^+^ (active) T cell populations in INDO-treated tumors, while a separate CODEX image analysis showed increased cytolytic/exhausted CD8^+^ ratios in 6 Gy + INDO treated tumors (Figure 4A). CODEX spatial distribution analysis also revealed increased CD8^+^ T cell penetration into the tumor core in INDO-treated tumors (Figure 4B). In contrast, control tumors showed sparsely populated CD8^+^ T cells (Figure 4A) that were largely restricted to the tumor margins (Figure 4B). While a proportional increase in lymphocytes was observed overall, the distribution of CD8^+^ T cells in the tumor core in response to INDO treatment was reminiscent of fully inflamed TNBC tumors shown in Figure 2A, this fully inflamed phenotype may in part account for the augmented tumor growth delay of the primary lesion (Figure 3A). Next, T cell polarization was examined in control and treated tumors using RNAScope analysis of IFN-γ and Granzyme B (GrnzB) secreted by cytolytic CD8^+^ T cells, as well as the immunosuppressive biomarker IL-10. When compared with untreated controls, Figure 5 shows increased IFN-γ in all treated tumors. GrnzB was enhanced in 6 Gy–treated tumors, while INDO and 6 Gy + INDO combination treatment trended higher (Figure 5). In contrast, when compared with control, the expression levels of immunosuppressive IL-10 did not change significantly in treated tumors. Interestingly, spatial analyses revealed that CD8^+^ T cells in treated tumors were surrounded by active CD4^+^ T cell and CD19^+^ B cell phenotypes (compare Figure 4B and Supplemental Figure 5). Given that tumor-infiltrating B cells can secrete apoptosis-inducing IgG antibodies as well as function as antigen-presenting cells that prime CD4^+^ and CD8^+^ T cells, this spatial lymphoid distribution is consistent with tertiary lymphoid structures, which may suggest a unique orthogonal pattern of immune cell trafficking leading to improved therapeutic efficacy (34, 35).

### COX inhibition augments cGAS/STING1/type I IFN in irradiated 4T1 tumors.

**Figure 6:**
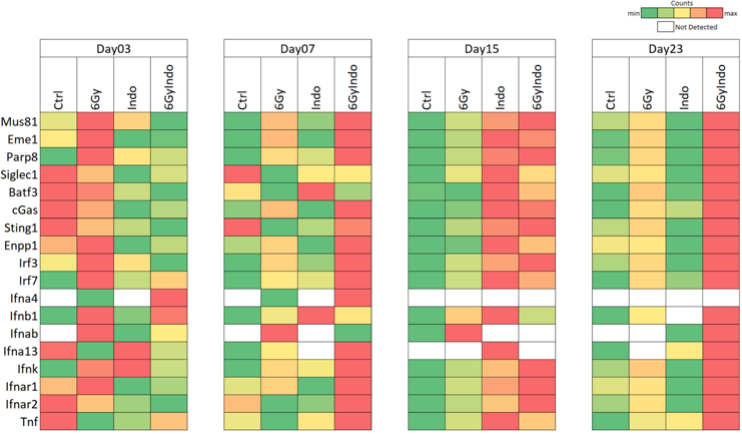
Increased cGAS/STING1 in 6 Gy + INDO–treated tumors. Heatmaps analysis of individual genes related to the cGAS/STING pathway leading to augmented type I IFN in 6 Gy–, INDO-, and 6 Gy+INDO–treated samples. The green-to-red (low-to-high) color scale indicates the number of transcript counts. White boxes indicate no transcripts were found. Prior to heatmap development in Microsoft 64-bit Excel 365, transcript counts were normalized using the default “counts per million + 0.0001” method in the Partek Flow software (Build 10.0.22.0428).

**Figure 7:**
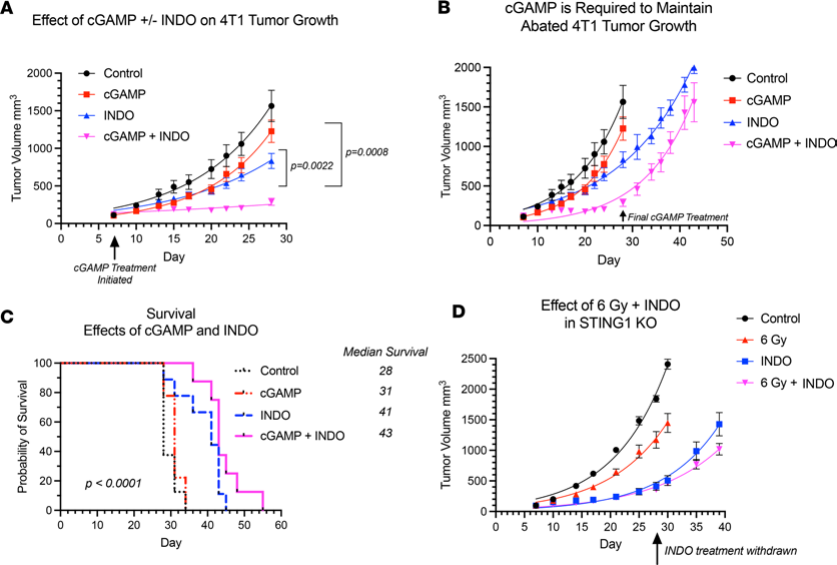
STING agonist cGAMP + INDO abates 4T1 tumor growth. (**A**) The STING agonist cGAMP was administered intratumorally (5 μg/50 μL in endotoxin-free H_2_0) 2 times per week for 3 weeks beginning on day 7 as indicated by the arrows. INDO administration in the drinking water also began on day 7 and was present continuously throughout the experiment. The cGAMP + INDO combination treatment completely abated tumor growth. Two-way ANOVA with Tukey’s multiple-comparison test was used. (**B**) Tumor growth in cGAMP + INDO resumed after cGAMP treatment stopped on day 29. (**C**) cGAMP + INDO combination treatment improved median survival when compared with cGAMP treatment alone. Statistical Log-rank (Mantel-Cox) test was used. (**D**) INDO treatment promoted antitumor effects independent of radiation in STING-KO mice indicating the importance of local tumor STING response versus systemic STING depletion in mice.

**Figure 8:**
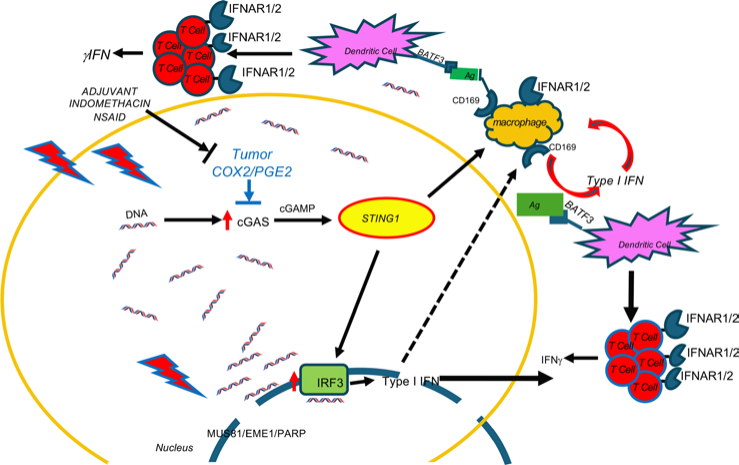
Indomethacin augments cytoplasmic tumor DNA accumulation and downstream induction of cGAS/STING1, and type I IFN in treated tumors. Radiation + indomethacin for COX2 inhibition increases cytoplasmic tumor DNA accumulation through MUS81/EME1/PARP. Accumulated cytoplasmic tumor DNA then induces cGAS/STING, type I IFNs, IFNAR, and increases cytolytic T cells.

In addition to T cells, other CD45^+^ immune cells were examined (Supplemental Figure 6), including DC, macrophages, and NK cells that also express CD8. Increased trends in CD11c^+^CD8^+^ DCs and CD11c^+^CD8^+^CD169^+^ cells were observed (Supplemental Figure 7). Also, elevated CD169^+^MHCII^+^, F4/80^+^CD169^+^, CD11b^+^CD169^+^, and MHCII^+^CD8^+^CD3^–^ macrophage populations were observed (Supplemental Figure 7). CD169, a biomarker of STING, is involved in antigen presentation, cross-priming, and expansion of CD8^+^ T cells (36–39). This process occurs when CD169^+^ macrophages bind to sialic acids on CD8α^+^ DCs (36, 38). Given that increased CD169^+^ macrophage populations as well as increased trends in CD11c^+^CD8^+^ DC and CD11c^+^CD8^+^CD169^+^ cells were observed (Supplemental Figure 7), RNA-Seq gene expression was analyzed for a STING signature. Figure 6 and Supplemental Figure 8 show increased expression of MUS81/EME1/PARP biomarkers known to induce cGAS/STING through the cytosolic accumulation of tumor DNA (40, 41). In addition, elevated downstream cGAS/STING/type I IFN expression in 6 Gy ± INDO–treated tumors was also observed, and it began as early as day 3 and persisted through day 23 after IR (Figure 6 and Supplemental Figure 8). This cGAS/STING/type I IFN pathway is supported by increased IFN and IFN response gene expression and altered immunosuppressive gene expression shown in Supplemental Figure 9. Because these results strongly implicate a role of cGAS/STING in the 6 Gy + INDO antitumor immune response, we examined the antitumor effects of STING agonist cGAMP in the presence and absence of INDO in 4T1 tumor–bearing mice. The STING agonist cGAMP was administered 2 times per week for 3 weeks beginning on day 7 as indicated by the arrows in Figure 7A. INDO administration in the drinking water also began on day 7 and was present continuously throughout the experiment. Importantly, the cGAMP + INDO combination treatment completely abated 4T1 tumor growth (Figure 7A) in a remarkably similar fashion to that observed in 6 Gy + INDO–treated mice (Figure 3A). However, tumor growth resumed after cGAMP treatment stopped (Figure 7B), marking abated tumor immune surveillance induced by cGAMP (42). In addition, cGAMP + INDO combination treatment increased median survival when compared with cGAMP alone (Figure 7C). These results were further examined in 4T1 tumor–bearing STING1-KO mice. Despite the experiment being done under conditions of *systemic STING1 depletion* in the STING1-KO mouse, an effective antitumor response was achieved due to the localized response to focused irradiation of the 4T1 tumor that still expressed cGAS/STING1 (Figure 6, Figure 7D, and Supplemental Figure 8). Also, the cGAS/STING1 pathway can promote both pro- and antitumor responses (43), where STING1 signaling has a role in the development of many leukocyte populations, including Treg immunosuppressive phenotypes, which are not induced in the STING1-KO mouse. Moreover, limited tumor growth has been shown in LLC tumor–bearing STING-KO mice when compared with WT controls (44). Together, our results with focused 4T1 tumor irradiation or local administration of cGAMP implicate the importance of the localized induction of STING signaling within the tumor that can be augmented by COX inhibition to enhance antitumor immune response. In addition, 4T1 tumor cells are known to express GM-CSF, which is elevated along with the chemokine CCL2 in 6 Gy + INDO–treated mice (Supplemental Figure 10). This could also contribute to the resumed tumor growth of combination-treated mice, thus providing additional therapeutic targets for improved clinical responses. Importantly, an undesired effect of local radiotherapy involves increased release of GM-CSF/CCL2 and the formation of premetastatic niches by recruitment of M-MDSCs into the lungs of 4T1 tumor–bearing mice (45). Together, this work suggests that the 6 Gy + INDO treatment mediates a temporal progression of CD8^+^ T cells and antigen presenting cells with proinflammatory and antitumor function that is in part mediated by STING mechanisms, as summarized in Figure 8. This is supported by the GSE37751 database (https://www.ncbi.nlm.nih.gov/geo/query/acc.cgi?acc=GSE264712) probability of survival stratifying for the effects of COX2 in patients with TNBC with high versus low STING1 expression. When compared with high tumor COX2, low tumor COX2 expression increased the probability of survival in these patients (HR = 0.2857, *P* = 0.0426; Supplemental Figure 11).

## Discussion

Improved clinical outcomes associated with many cancers including TNBC directly correlate with elevated infiltrating CD8^+^ T cells, implicating the importance of antitumor immune response for improved treatment efficacy and survival (46). These findings have been extended by a recent study showing that, in addition to increased infiltration and density, the spatial localization of CD8^+^ T cells was critical for improved survival of patients with TNBC (2). Tumor immune deserts (<100 CD8^+^ T cells per mm^2^) exhibited increased expression of immunosuppressive B7-H4 and fibrotic signatures predictive of poor survival (2). In addition, stroma-restricted CD8^+^ T cells were associated with an immunosuppressed TME through elevated cholesterol signatures TGF-β, IL17, and TANs, and these elevated signatures predicted poor survival. In contrast, elevated CD8^+^ T cell penetration into the tumor epithelium was defined as a fully inflamed tumor and correlated with increased GrnzB, type I IFN, IDO, and PD-L1 expression that predicted improved survival. Moreover, type I IFN and cholesterol biosynthesis pathways were shown to negatively regulate one another. Given that type I IFN signatures were associated with CD8^+^ T cell penetration into the tumor core while cholesterol signatures were associated with the restriction of CD8^+^ T cells in tumor stroma, these observations implicate a key role of the spatial configuration of CD8^+^ T cells during polarization of the tumor immune microenvironment that was further validated in a cohort of 579 patients with TNBC (2). These results suggest that CD8^+^ T cell orientation and increased type I IFN–associated antitumor immunity improves clinical outcomes in TNBC (2).

The inducible isoform COX2 catalyzes the first step in prostanoid synthesis and is expressed at high levels in many tumors, including TNBC (13, 16, 18, 22). While COX2 is known to promote angiogenesis, drug resistance, and metastasis, it also contributes to immune evasion and resistance to cancer immunotherapy (11, 28). The COX2/PGE2/EP signaling pathway suppresses DCs and NK cells and inhibits T cell production and responsiveness of IL-2 that limits both activation and expansion of cytolytic T cells and promotes tumor immune evasion, suggesting that COX2 blockade could restore tumor immune surveillance (11, 16). Toward this end, NSAIDs have been used in both preventative and adjuvant anticancer applications (47). As adjuvant, the benefits of NSAIDs have been shown when used in conjunction with therapeutic radiation for treatment of prostate and other cancers (22, 47). Herein, we extend these observations by showing that 6 Gy radiation combined with the NSAID INDO augmented cGAS/STING1, type I IFNs, and cytolytic CD8^+^ T cells; this augmentation appeared to restore immune surveillance, limit tumor growth and metastatic burden, and improve survival in the aggressive 4T1 TNBC model. In addition, when combined with INDO, the STING agonist cGAMP abated tumor growth (Figure 7A) in a remarkably similar fashion as the 6 Gy + INDO combination (Figure 3A) and was consistent with augmented antitumor effects of celecoxib/cyclic diadenyl monophosphate (celecoxib/CDA) combination treatment of mice with Lewis lung carcinoma (48). In our work, cGAMP withdrawal was the limiting factor (Figure 7B) as tumor volumes reached the allowable limit after cGAMP administration was stopped and mice had to be euthanized; despite this, the cGAMP/INDO combination treatment improved survival when compared with cGAMP treatment alone (Figure 7, B and C). Despite systemic depletion in STING1-KO mice, COX inhibition by INDO effectively limited tumor growth in irradiated 4T1 tumors, emphasizing the importance of the localized STING response within the tumor (Figure 7D). STING1 signaling also promotes leukocyte populations, including Treg immunosuppressive phenotypes, as well as IL-10, IDO, and COX2 immunosuppressive mediators, which are not induced under conditions of systemic STING depletion in the STING1-KO mouse. Therefore, the results herein suggest the importance of systemic depletion versus localized STING induction for restored antitumor immune surveillance in 4T1 tumor–bearing mice (44). Moreover, our results show synergistic effects of radiation/NSAID combination treatment that augmented the localized tumor induction of STING signaling and type I IFNs when compared with either treatment alone. Together, these results suggest therapeutically beneficial effects of localized STING antitumor response following radiation/NSAID combination treatment and are supported by GSE37751 database probability of survival examining the influence of tumor COX2 on STING1 (Supplemental Figure 11).

Type I IFNs mediate diverse antitumor effects, including immune surveillance of precancerous lesions, inhibitory effects against the disease progression of established tumors by augmented cell cycle arrest and apoptosis, and abated invasion and metastasis of established tumors (49). During immune surveillance, type I IFN generated by immunogenic precancerous cells activates immune cells, including DC, macrophages, and cytolytic T cells that induce IFN-γ for elimination of the neoplastic lesion (49). A plethora of studies have shown that conventional therapies, including chemo and therapeutic radiation, promote cytosolic tumor DNA accumulation and cGAS/STING-mediated type I IFN antitumor immune responses (49–51). Moreover, chimeric antigen receptor (CAR) T cell–based therapies have demonstrated the requirement of IFNAR1/2 receptor signaling for both survival and cytolytic activity of CAR T cells (52). While these and other studies have demonstrated the importance of type I IFN for anticancer immunity and improved patient survival, most tumors exhibit dysregulated type I IFN signaling, leading to protumor resistance mechanisms (53). Interestingly, low, basal levels of type I IFN expressed in cancer cells have accounted for enhanced immunity in response to immunotherapies that is driven by DNA leakage and cGAS/STING (54–56). Indeed, most tumor-relevant type I IFNs are induced by cytosolic or endolysosomal sensing of nucleic acids, where DNA that has leaked from the nucleus or mitochondria of tumor cells can induce cGAS/STING signaling (50). In addition to the direct generation of DNA fragments from double stranded (ds) DNA breaks caused by radiation, leaked DNA can arise from mutations in oncogenes and tumor suppressor genes causing dysregulated cancer cell replication and stalling of replication forks. A replication stress response is then triggered, which restarts the stalled replication forks (40). During this process, the DNA endonuclease MUS81/EME1 complex and PARP-dependent DNA repair pathways mediate shedding and cytosolic accumulation of genomic tumor DNA (40, 57). The accumulation of cytosolic DNA induced STING signaling, type I and II IFN, cytolytic T cell immune responses, and macrophage-dependent tumor cell rejection (40). Moreover, CD169-expressing macrophages promote DC antigen presentation during CD8^+^ T cell cross-priming and activation, and DC are thought to be critical for type I IFN antitumor responses (36–39). Herein, gene expression analysis showed the induction of MUS81/EME1/PARP, CD169, cGAS, STING1, type I and II IFN, and type I IFN receptors IFNAR1/2 in INDO and 6 Gy + INDO–treated tumors on days 15 and 23 after IR (Figure 6 and Supplemental Figure 8). In addition, cGAMP + INDO combination abated tumor growth in a remarkably similar fashion as 6 Gy + INDO treatment (compare Figure 3A and Figure 7). The flattened tumor growth associated with cGAMP + INDO treatment demonstrated in Figure 7A required cGAMP as tumor growth resumed upon withdrawal of the STING agonist (Figure 7B). Together, these results show that the 6 Gy + INDO combination treatment restored tumor immune surveillance against aggressive 4T1 tumors, which involved at least in part enhanced antitumor innate and adaptive immune responses through cGAS/STING/type I IFN signaling.

Herein, we provide evidence demonstrating that INDO-mediated COX2 inhibition augments MUS81-mediated cytoplasmic tumor DNA accumulation and cGAS/STING/type I IFN, which restored tumor immune surveillance via potent antitumor innate and adaptive immune responses in irradiated 4T1 TNBC tumors. Together, these results demonstrate immune changes that occur through abated COX2 signaling, which promotes altered spatial organization of M1/Th1 immune phenotypes and augmented antitumor response. Whole tumor RNA-Seq revealed important changes beginning as early as day 3 after IR that persisted throughout the experiments, showing that 6 Gy + INDO treatment supports increased cGAS/STING signaling and augmented type I IFN expression including IFN-αβ, IFN-β1, IFN-α4, and IFN-α13 as well as increased IRF3 and type I IFN receptors IFNAR1/2 (Figure 6 and Supplemental Figure 8). In addition, increased IRF7 (Figure 6 and Supplemental Figure 8), which is a master regulator in the induction of type I IFN-β such as IFN-αβ (58), was also observed. These results highlight major changes in the spatial immune landscape of 6 Gy + INDO-treated tumors that augments antitumor immunity and reverses tumor immune suppression. Altered spatial immune landscapes were confirmed in TNBC where high CD8^+^ T cell penetration was observed in COX2_lo_ fully inflamed tumor epithelium. In contrast, stroma restricted CD8^+^ T cells and immune deserts were found in COX2_hi_ TNBC tumors (Figure 2). Importantly, COX2/CD8 ratios were elevated in deceased patients with TNBC (Figure 2H) and elevated tumor COX2 expression reduced radiation therapeutic efficacy (Figure 1). These results strongly implicate COX2 as a key mediator of immunosuppression, limited adaptive immunity, and poor clinical outcomes. Early studies show that type I IFN can provide an important antitumor tool that improves efficacies of standard-of-care therapies. However, systemic administration of these IFNs and other cytokines has severe side effects that limit their clinical use (49). Here, we show that NSAIDs and focused tumor irradiation can provide a localized tumor-specific cGAS/STING-mediated generation of type I IFN, as summarized in Figure 8, which had profound effects on the spatial localization of CD8^+^ T cells, favoring antitumor immune response in aggressive 4T1 murine tumors. Targeting COX2 in combination with radiation enhances local control of the primary lesion by augmented M1/Th1 immune mediators, restored immune surveillance, and reduced metastatic burden. Given the challenges associated with the clinical administration of STING agonists in cancer and immune therapy and the clinical availability of COX inhibitors, these results suggest that therapeutic radiation and COX inhibition may provide a readily available approach for the localized induction of STING antitumor response that could improve clinical outcomes in patients with aggressive TNBC tumors.

## Methods

## Sex as a biological variable

Pearson correlation coefficients for the incidence rates of female versus male breast cancer have been reported (59). Male breast cancer rates were generally less than 1 per 100,000 man years, in contrast to the much higher rates of female breast cancer of 122. The differences in both incidence rates and time trends between males and females may reflect sex differences in underlying risk factors, including differences in ducts and lobules and the absence of p53 mutation (60). While most males are ER^+^ and ductal carcinoma in situ (DCIS) represents 10% of male breast cancers, TNBC is less frequent with poorer prognosis due to higher histopathological grade (61). Given these low occurrences in males, female patients with TNBC were examined for the effects of tumor NOS2 and COX2 expression on radiation therapeutic efficacy.

### Immunophenotyping of tumors by flow cytometry

Tumors were dissociated by mechanical dissociation (Miltenyi GentleMACS) in lysis buffer containing Collagenase and DNase in 5% RPMI. RBCs were removed by incubating in ACK lysis buffer and washing with phosphate buffered saline (PBS). Cells were counted, and equal numbers of cells were stained with the Live/Dead Aqua reagent (Amcyan) (1:1,000) in PBS for 30 minutes followed by PBS wash and 20 minutes at 4°C with Fc blocker (1:200) in sorter buffer (1%FBS, 1 mM EDTA in PBS). The cells were then stained with a panel of fluorophore-tagged antibodies against various immune cell markers including CD45-FITC (clone 30-F11, 103107), CD3-BV785 (clone OKT3, 317329), CD4-PECy7 (clone RM4-5, 100527), CD8a-PerCPCy5.5 (clone 53-6.7, 100733), CD19-BV605 (clone HIB 19, 302243), Tim3-APC (clone F38-2E2, 345011), CD62L-PE (clone MEL-14, 104407), CD45-BV605 (clone HI30, 304041), CD11b-PerCPCy5.5 (cloneM1/70, 101227), CD11c-APC Cy7 (clone N418, 117323), F4/80-APC (clone BM8, 123115), Ly6G-BV711 (clone 1A8, 127643), Ly6C-PE Cy7 (clone HK-1.4, 128017), CD206-FITC (clone 15-2, 321103), PD-L1-PE (clone MIH2, 393607), and MHCII (MHC1A/1E)-BV421 (clone M5/114.15.2, 107631), from BioLegend. Samples were incubated for 20 minutes at 4°C, washed, and read on a flow cytometer. Respective unstained cells and fluorescence minus one (FMO) controls were used to set the positive gates during acquisition. Samples were acquired using the low/medium flow rate setting on the BD LSRII Sorp flow cytometer, normalized to tumor weight, and analysis was performed using FlowJo software.

### RNA-Seq of bulk tumor

In brief, fresh frozen tissue samples were homogenized in the presence of TRIzol (Thermo Fisher Scientific) and further purified with affinity column (RNeasy Mini Kit, Qiagen) following the manufacturer’s recommendation. The RNA quality following extraction was checked in a bioanalyzer, and only samples with a RNA integrity number (RIN) larger than 6 were used to make the RNA-Seq library prep. Sample libraries were prepped with the Illumina Stranded Total RNA Prep, and paired-end sequencing was performed according to the manufacturer protocol and sequenced in a NovaSeq 600 sequencing system. Reads of the samples were trimmed for adapters and low-quality bases using Cutadapt. Sequencing data were exported and then uploaded to the Partek Flow server for subsequent sample normalization and QC steps using the built-in RNA-Seq Data Analysis workflow. Differentially expressed gene lists were generated with the Partek GSA algorithm, which applies multiple statistical models to each individual gene in order to account for each gene’s varying responses to different experimental factors and to different data distributions. Prior to heatmap development in Microsoft 64-bit Excel 365, transcript counts were normalized using the default “counts per million + 0.0001” method in the Partek Flow software (Build 10.0.22.0428). All data were normalized to internal housekeeping genes. A 2-fold cutoff and *P* value < 0.05 filter was applied to finalize the gene lists.

### CODEX analysis

The CODEX fixation and staining protocol was performed according to Akoya User Manual; revision B.0. Square (22 × 22 mm) glass coverslips (72204-10, Electron Microscopy Sciences) were pretreated with L-Lysine (MilliporeSigma) overnight at room temperature. Coverslips were rinsed in distilled water, dried, and stored at room temperature. Fresh frozen tissue blocks were sectioned (10 μm) on treated coverslips and stored in a coverslip storage box (Qintay) at –80°C until further use. CODEX antibodies, reagents (including those for conjugation of custom antibodies), and instrumentation were purchased from Akoya Biosciences). Antibodies labeled for CODEX are a mix of commercial and custom antibodies to identify different immune cell phenotypes, which included CD279 (clone RMP1-30, 109101, BioLegend), CD86 (clone GL1, 14-0861-82, Invitrogen), Ki67 (clone B56, 4250019, Akoya Biosciences), E-cadherin (clone 24E10, 3195 Cell Signaling Technology), CD19 (clone B56, 4250019, Akoya Biosciences), CD31 (clone MEC13.3, 4250001, Akoya Biosciences), CD49f (clone GoH3, 4550102, Akoya Biosciences), vimentin (clone D21H3, 5741, Cell Signaling Technology), F4-80 (clone T45-2342, 565409, BD Pharmingen), αSMA (clone 1A4, 14-9760-82, Invitrogen), CD44v6 (clone 9A4, BMS145, eBiosciences), Ly6C (clone HK1.4, 128001, BioLegend), NOS2 (clone EPR16635, ab213987, Abcam), CD206 (clone MR5D3, MA5-16871, Invitrogen), CD25 (clone PC61, 102007, BioLegend), CD11c (clone N418, 4550108, Akoya Biosciences), CD274 (clone MIH1, 14-5983-82, Invitrogen), CD44 (clone IM7, 4250002, Akoya Biosciences), CD24 (clone M1/69, 4150014, Akoya Biosciences), MHCII (clone M5/114.15.2, 4250003, Akoya Biosciences), CD3 (clone 17A2, 4550109, Akoya Biosciences), CD90.2 (clone 30-H12, 4150001, Akoya Biosciences), CD5 (clone 53-7.3, 4250007, Akoya Biosciences), CD71 (clone R17217, 4550111, Akoya Biosciences), CD45 (clone 30-F11, 4150002, Akoya Biosciences), CD4 (clone RM4-5, 4250016, Akoya Biosciences), CD169 (clone 3D6.112, 4550100, Akoya Biosciences), CD38 (clone 90, 4150013, Akoya Biosciences), CD8a (clone 53-6.7, 4250017, Akoya Biosciences), Ly6G (clone 1A8, 4550110, Akoya Biosciences), and CD11b (clone M1/70, 4150015, Akoya Biosciences). Tissue sections were stained with an antibody cocktail consisting of the above antibodies diluted 1:200 or 1:400. CODEX assays were performed according to the manufacturer’s recommendations. Each CODEX cycle contains 4 fluorescent channels (3 for antibody visualization and 1 for DAPI nuclear stain). Fluorescent oligonucleotide plates were prepared in black 96-well plates for image acquisition. For each cycle, up to 3 fluorescent oligonucleotides (5 μL each) were added to a final volume of 250 μL of buffer (containing DAPI nuclear stain). For blank (empty) cycles, 5 μL of buffer was substituted for fluorescent oligonucleotides. Plates were sealed and kept at 4°C until use. For imaging, the CODEX coverslip was mounted onto a custom-designed plate holder and securely tightened onto the stage of a Keyence BZ-X810 inverted fluorescence microscope. Cycles of hybridization, buffer exchange, image acquisition, and stripping were then performed using an Akoya CODEX microfluidics instrument. Briefly, the microfluidics-microscope combined instrument performs hybridization of the fluorescent oligonucleotides in a hybridization buffer, imaging of tissues in CODEX buffer, and stripping of fluorescent oligonucleotides in the stripping buffer. CODEX multicycle automated tumor imaging was performed using a CFI Plan Apo 20×/0.75 objective (Nikon). The multipoint function of the BZ-X viewer software (BZ-X ver. 1.3.2, Keyence) was manually programmed to align with the center of each tumor and set to 10 *Z* stacks. DAPI nuclear stain (1:600 final concentration) was imaged in each cycle at an optimized exposure time of 10 ms. The respective channels were imaged in the automated run using optimized exposure times. Raw TIFF images produced during image acquisition were processed using the CODEX image processer. The processer concatenates *Z* stack images, performs drift compensation based on alignment of nuclear stain across images, and removes the out-of-focus light using the Microvolution deconvolution algorithm (Microvolution). The processer also corrects for nonuniform illumination and subtracts the background and artefacts using blank imaging cycles without fluorescent oligonucleotides. The output of this image processing was tiled images corresponding to all fluorescence channels and imaging cycles that were then visualized and analyzed using HALO software (Version 3.3.2541.383, Indica Labs Inc.). Segmentation of cells was performed using the nuclear channel, and the cell cytoplasm was defined as a fixed width ring around each nucleus. Nuclear segmentation settings were optimized by visual verification of segmentation performance on random subsets of cells aiming to minimize oversegmentation, undersegmentation, detected artefacts, and missed cells. Cell type annotation and differential marker analysis cell populations were gated as follows. All nucleated cells were first identified by positive nuclear signals. Cell phenotypes were defined based upon biomarker expression as judged by expert visual inspection.

### RNAScope

RNAScope (Advanced Cell Diagnostics [ACD] Bio) fluorescence in situ hybridization (FISH) was performed on 5 μm cryosections of samples using the RNAScope Multiplex Fluorescent Reagent Kit V2 (ACD) according to the manufacturer’s instructions. In brief, RNA-FISH was performed on all tumor samples by the Molecular Pathology Laboratory at the National Cancer Institute at Frederick. Serial cryosections of each sample were also processed for H&E and CODEX staining as well as RNA-Seq analyses. Histopathology was performed by a board-certified veterinary pathologist. RNAScope target probe staining was performed using a Leica Biosystems Bond Rx automated IHC/FISH slide staining system (Leica Biosystems). For each set of RNA in situ hybridization probes stained, an RNAScope positive control and negative control probe were included to serve as an assay control. All target RNAScope probes were tested in a separate pilot study for the validation of probe specificity and localization. Digital fluorescent images (20×) were acquired on an Aperio ScanScope FL Scanner (Leica Biosystems), and object cell fluorescence intensity for each probe was quantified with HALO Imaging Software (Indica Labs). A classifier was built to distinguish between viable and necrotic tumor areas for each sample. Poorly stained areas were excluded; then, the fluorescent channels were adjusted by eye for each probe, and tissues were quantified. All data were exported to Excel (Microsoft) and Prism (GraphPad) for subsequent data and statistical analyses.

### Image analysis using HALO

CODEX and InSituPlex images were analyzed using HALO V3.3 (Indica Labs) available through the NCI HALO Image Analysis Resource. Registered and stacked InSituPlex images and processed CODEX images were fused to generate afi composite images for analysis. Segmentation of cells was performed using the nuclear channel and the cell cytoplasm was defined as a fixed width ring (2 μm) around each nucleus. Nuclear segmentation settings were optimized by visual verification of segmentation performance on random subsets of cells aiming to minimize the number of oversegmentation, undersegmentation, detected artefacts, and missed cells. Signal thresholding for each individual signal was defined based upon biomarker expression as judged by expert visual inspection independently on each image. Cellular phenotypes were set based on the combination of signals described in Supplemental Table 2 using HALO and/or positive and negative criteria. Regions of viable tumor, stroma, and necrosis were annotated manually on H&E-stained slides. The H&E annotations were fused with Ultivue and COX2-stained images to spatially localize COX2 expression and CD8^+^ T cells relative to viable tumor or stroma.

### Genome Expression Omnibus

The GSE37751 breast cancer data were obtained from the Genome Expression Omnibus (GEO) public data repository (https://www.ncbi.nlm.nih.gov/geo/query/acc.cgi?acc=GSE264712). The R software (version 4.2) was used to extract gene expression data from TNBC samples for subsequent analysis. Briefly, COX2 and STING1 gene expression and associated survival data were extracted and processed together. The data set was divided into 2 subsets by the median COX2 expression value (high versus low). These COX2 subsets were then stratified for STING1 gene expression median values. The associated survival data from these subsets (COX2_hi_: STING1_lo_ versus STING1_hi_; COX2 _lo_: STING1_lo_ versus STING1_hi_) were exported to Prism (v10), and probability of survival was plotted. The *P* values were determined using log-rank (Mantel-Cox) test, and HRs were calculated using Mantel-Haenszel test.

### Statistics

Kaplan-Meier survival analysis was performed using the Stata/SE 14 (Stata Corp.) statistical software package. Median and mean follow-up times for disease-free survival were 65 months and 57 months, respectively (range, 1–186 months). A total of 60 of 200 patients experience a recurrence during this time. Kaplan-Meier curves and log-rank test for equality of the survival function was used for univariate survival analysis. Cox regression analysis was used to calculate HRs and to perform multivariable analysis. For animal experiments 2-way ANOVA with Tukey’s multiple-comparison test was employed to assess significance of tumor growth data. All other analyses employed Welch’s, Mann-Whitney *U* tests, or 1-way ANOVA with Dunnett’s multiple-comparison test, or the nonparametric Kruskal-Wallis test for comparison of 2 or more groups of equal or different sample sizes, to assess statistical significance of mean values using the GraphPad Prism software. Results are presented as mean ± SEM, and *P* ≤ 0.05 was considered significant.

#### Kaplan-Meier survival analysis.

A subset of 210 TNBC specimens from a larger TNBC cohort (26) arrayed on a tissue microarray were stained for COX2 expression by IHC as previously described (12) and for NOS2 expression as previously described (62). Paraffin-embedded (*n* = 210) tumor specimens were obtained from patients with breast cancer diagnosed with TNBC (confirmed ER/PR/HER2^–^) at Galway University Hospitals between 1999 and 2016. Areas of tumor were identified by the pathologist, and a tissue microarray was constructed. Clinical and pathological information were obtained from medical oncology and pathology reports. Disease staging was performed according to the tumor-node-metastasis (TNM) system of the American Joint Committee on Cancer/Union Internationale Contre le Cancer (AJCC/UICC). The Nottingham system was used to determine tumor grade. Disease-free survival was defined as no recurrence at the local site (breast), at the regional site (lymph nodes), or at distant sites. Patients who had distant metastasis at diagnosis were excluded from recurrence free survival. High versus low COX2 expression was defined based on IHC intensity and distribution scores where IHC intensity received scores of 0 to 3 if the staining was negative, weak, moderate, or strong. The distribution received scores of 0 to 4 if the staining distribution was <10% positive cells, 10%–30%, >30%–50%, >50%–80%, and >80%. A sum score was then divided into 4 groups as follows: (a) negative = 0–1, (b) weak = 2–3, (c) moderate = 4–5, and (d) strong = 6–7. The effect of COX2 expression on radiation therapeutic efficacy was evaluated in patients who had received fractionated x-ray irradiation doses totaling 50 Gy.

#### IHC analysis of patient tumor sections.

Tumor specimens were obtained from patients with breast cancer recruited at the University of Maryland (UMD) Medical Center, the Baltimore Veterans Affairs Medical Center, Union Memorial Hospital, Mercy Medical Center, and the Sinai Hospital in Baltimore between 1993 and 2003. Breast tumor COX2 expression was analyzed previously by IHC using a 1:50 diluted COX2 antibody (BD Biosciences, clone 33; no. 610204). COX2 expression was scored by a pathologist; scores of negative to weak (scores 1 and 2) or moderate to strong (scores 3 and 4) were categorized as low or high, respectively (13). Herein, COX2 expression analyzed by fluorescence staining was performed on the Leica Biosystems Bond RX autostainer using the Bond Polymer Refine Kit (Leica Biosystems, DS9800), with omission of the PostPrimary reagent, DAB, and Hematoxylin. After antigen retrieval with EDTA (BOND Epitope Retrieval 2, Leica), sections were incubated for 30 minutes with Cox2 (Cell Signaling Technology, 12282, 1:100), followed by the polymer reagent and OPAL Fluorophore 520 (Akoya). The original IHC and COX2 fluorescence staining results were generally consistent.

Formalin-fixed paraffin embedded (FFPE) tissue sectioned at 4 μm and mounted on SuperFrost Plus slides were stained with a FixVUE Immuno-8 Kit (formerly referred to as UltiMapper kits, Ultivue Inc.; CD8, PD-1, PD-L1, CD68, CD3, CD8, FoxP3, and panCK/SOX10 cocktail) using the antibody-conjugated DNA-barcoded multiplexed immunofluorescence (mIF) method (63). The kits include the required buffers and reagents to run the assays: antibody diluent, preamplification mix, amplification enzyme and buffer, fluorescent probes and corresponding buffer, and nuclear counterstain reagent. H&E and mIF staining was performed using the Leica Biosystems BOND RX autostainer using FixVUE (UltiMapper) protocol. First, FFPE tissue sections were baked vertically at 60°C–65°C for 30 minutes to remove excess paraffin prior to loading on the BOND RX. The BOND RX was used to stain the slides with the recommended FixVUE (UltiMapper) protocol. During assay setup, the reagents from the kit were prepared and loaded onto the autostainer in Leica Titration containers. Solutions for epitope retrieval (ER2, Leica Biosystems, AR9640) and BOND Wash (Leica Biosystems, AR9590), along with all other BOND RX bulk reagents, were purchased from Leica. During this assay, the sample was first incubated with a mixture of all 8 antibody conjugates; next, the DNA barcodes of each target were simultaneously amplified to improve the sensitivity of the assay. Fluorescent probes conjugated with complementary DNA barcodes were then added to the sample to bind and label the first round of 4 targets; a Round 1 fluorescent image was then acquired. Next, a gentle signal-removal step was used to remove the fluorescent probes of the first set of markers before adding the fluorescent probes specific for the second set of 4 markers; then imaging the slide was performed a second time to acquire the Round 2 fluorescent image. There was no need for quenching, bleaching, or other means to minimize signal between rounds. Before each round of imaging, the stained slides were mounted in Prolong Gold Anti-Fade mountant (Thermo Fisher Scientific, P36965 and coverslipped (Thermo Fisher Scientific, Fisherbrand Cover Glass 22 × 40 mm, no. 1.5). Digital IF images were scanned at 20× magnification. Round 1 and 2 images were coregistered and stacked with Ultivue’s UltiStacker software. The Ultivue Immuno8 FixVue Panel images used the following marker/fluorophore combinations with DAPI/FITC (CD8 Round 1 [R1]), CD3 R2, TRITC (PD-1 R1,CD4 R2), Cy5 (PD-L1 R1, FoxP3 R2), and Cy7 (CD68 R1, panCK/Sox10 R2). All digital images were then analyzed using HALO software (63).

#### In vivo studies.

Female BALB/c and C57BL/6 mice purchased from Charles River Laboratories were received at 7 weeks of age, housed 5 per cage, and given autoclaved food and water ad libitum. The mice were acclimated to the facility for 1 week prior to use. At 8 weeks of age, the mice were injected s.c. into the right hind limb with 200,000 4T1 (NCI-Frederick Cell Repository), EMT-6 (ATCC CRL-2755), or EO771 (ATCC CRL-3461) TNBC cells. Tumor measurements began 1 week after tumor cell injection, using a Vernier caliper and calculated in cubic millimeter volumes according to the following equation:

### ([short diameter]2 × long diameter)/2

Upon reaching tumor size of 100–200 mm^3^, tumor-bearing mice were divided into treatment groups of *n* = 10 or *n* = 5 for time course experiments. Tumor irradiation was accomplished by securing each animal in a specially designed Lucite jig fitted with lead shielding that protected the body from radiation while allowing exposure of the tumor-bearing leg. An XRAD320 x-ray cabinet (Precision X-Ray Inc.) using 1.5 mm Al/0.25 Cu/0.75 mm Sn filtration (300 KVp/12.5 mA) at a dose rate of 1.38 Gy/min was used as the x-ray source. Irradiated tumors received 1 dose of 6 Gy determined from the dose response curve shown in Supplemental Figure 3. This single dose yielded a growth delay that was markedly augmented by treatment with INDO. Importantly, in our study, a single dose of 6 Gy gave the same response as that shown by Vanpouille-Box et al., who administered a total of 30 Gy in 6 Gy dose fractions to 4T1 tumor–bearing mice (4). Given that our DER (DER 1.3) was consistent with that of Vanpouille-Box, along with added stress to the mice and the radiation sensitivity of T cells (30), we used the single-dose method. After tumor irradiation, the mice were returned to their cages and given INDO (30 mg/L) in the drinking water for the duration of the experiment. In a separate experiment, mice were treated with the STING agonist cGAMP (Invivogen) twice weekly for 3 weeks. Drinking water with and without INDO was changed every Monday, Wednesday, and Friday, and treatment continued for the duration of the experiment unless otherwise specified. Tumors were measured 3 times each week thereafter to assess tumor growth. Animals were euthanized when tumor growth approached the maximum allowable limit of 2,000 mm^3^. For the assessment of lung metastatic burden, lungs were fixed in Bouin’s solution and metastatic lesions were counted. Tumors were flash frozen, and serial 10 μm slices were sequentially obtained for RNA-Seq, CODEX, and RNAScope analyses.

### Study approval

Disease staging was performed according to the TNM system of the AJCC/UICC. The collection of tumor specimens and clinical and pathological information was reviewed and approved by the Galway University Hospitals Ethics Committee (approval no. CA1012). Informed consent was obtained from all patients. The collection of tumor specimens, survey data, and clinical and pathological information was reviewed and approved by the UMD IRB for the participating institutions. The research was also reviewed and approved by the NIH Office of Human Subjects Research (OHSR, no. 2248). Animal care provided at the NCI-Frederick Animal Facility was in accordance with the procedures outlined in the *Guide for the Care and Use of Laboratory Animals* (National Academies Press, 2011). The NCI-Frederick Animal Facility is accredited by the Association for Accreditation of Laboratory Animal Care International and follows the Public Health Service Policy for the Care and Use of Laboratory Animals.

### Data availability

Data are available at GEO accession no. GSE264712. All other data are available upon request to corresponding author. Supporting data, including values for all data points shown in graphs and mean values, are available in the Supporting Data Values file.

## Author contributions

Each author contributed as follows: LAR designed and conducted experiments, analyzed and interpreted data, and wrote the manuscript. RYSC designed and conducted experiments, analyzed and interpreted data, and wrote the manuscript. NK analyzed data. VS conducted experiments and analyzed data. DDB conducted experiments. DB conducted experiments and analyzed data. ALW analyzed data. AJW conducted experiments and analyzed data. CK analyzed data. WFH analyzed data. EFE provided pathology analysis. DOB conducted experiments. ACW conducted experiments. THD conducted experiments. MP analyzed data. RJK analyzed and interpreted data. SL analyzed and interpreted data. RJB analyzed and interpreted data. JR analyzed and interpreted data. STCW analyzed and interpreted data. SMH analyzed and interpreted data. JCC analyzed and interpreted data. AS conducted experiments and analyzed data. GMC analyzed and interpreted data. SAG provided cohort analyzed and interpreted data. SA provided cohort analyzed and interpreted data. SKA analyzed and interpreted data. DWM analyzed and interpreted data. SJL provided instrumentation, analyzed, and interpreted data. DAW conceptualized and designed experiments, analyzed and interpreted data, and wrote the manuscript.

## Supplementary Material

Supplemental data

Supporting data values

## Version 1 06/24/2024

Electronic publication
