# Supplementary material for: Adjuvant COX inhibition augments STING signaling and cytolytic T cell infiltration in irradiated 4T1 tumors
Source: JCI Insight. 2024 May 21;9(12):e165356. doi: 10.1172/jci.insight.165356 (PMC11383366; doi:10.1172/jci.insight.165356)
Supplement: Supplemental data [file jciinsight-9-165356-s225.pdf]

Supplemental Table I

Dose Enhancement Ratios

Treatment	Days to reach 500mm^3	DER
WT Control	16	1.00
WT 6 Gy	21	1.31
WT INDO	21	1.31
WT 6 Gy + INDO	30	1.88
WT Control	16	1.00
WT cGAMP	20	1.25
WT INDO	21	1.31
WT cGAMP + INDO	31	1.94
STING1ko Control	16	1.00
STING1ko 6 Gy	19	1.19
STING1ko INDO	29	1.81
STING1ko 6 Gy + INDO	31	1.94

Supplemental Table II

HALO Analysis Defined Phenotypes

$CD8^+$ Cytolytic T Cells	$CD45^+CD3^+CD8a^+CD279^-CD25^-$
$CD8^+$ Exhausted T Cell	$CD45^+CD3^+CD8a^+CD279^+CD25^-$
$CD4^+$ T Cell	$CD45^+CD3^+CD4^+CD25^-$
T_{reg}	$CD45^+CD3^+CD4^+CD25^+$
Dendritic Cell (DC)	$CD45^+CD11c^+$
Cross-Presenting DC	$CD45^+CD11c^+CD8^+$
Cross-Presenting DC-Macrophage	$CD45^+CD11c^+CD8a^+CD169^+$
$CD169^+MHCII^+$ Macrophage	$CD45^+CD169^+MHCII^+$
$CD169^+F4/80^+$ Macrophage	$CD45^+CD169^+F4/80^+$
$CD169^+CD11b^+$ Macrophage	$CD45^+CD169^+CD11b^+$
$CD8^+$ Monocytes	$CD45^+CD3^-CD8a^+MHCII^+$
$CD5^+CD19^+$ B Cell	$CD45^+CD5^+ CD19^+$
$CD19^+CD38^+$ B Cell Activation	$CD45^+CD5^+ CD19^+CD38^+$
$CD19^+CD25^+$ Mature B Cell	$CD45^+CD5^+ CD19^+CD25^+$

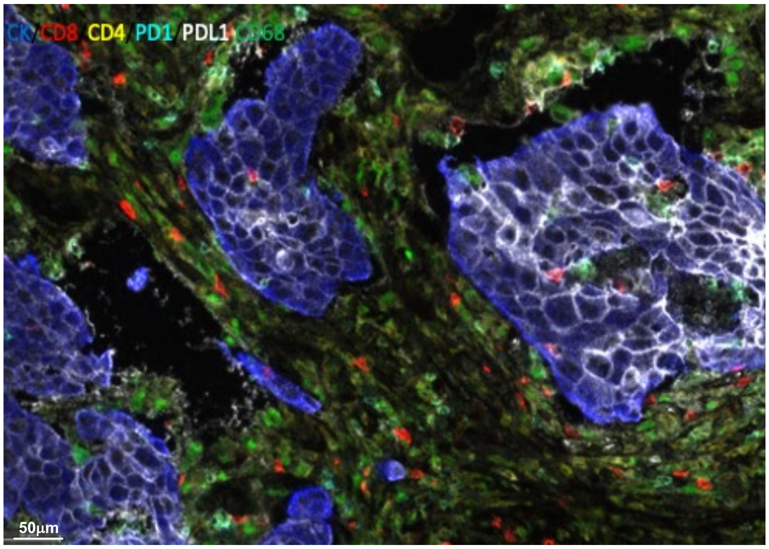

Supplemental Figure 1

Supplemental Figure 1

Multiplex imaging of tumor immune microenvironment. Ultivue multiplex imaging shows tumor immune microenvironment in a $COX2_{Hi}$ tumor. Biomarkers include CK/SOX1 (CK Blue)) tumor cell marker, CD8 (red), CD4 (yellow) T cell markers, PD1 (cyan), PDL1 (white) T cell exhaustion markers, and CD68 (green) macrophage marker.

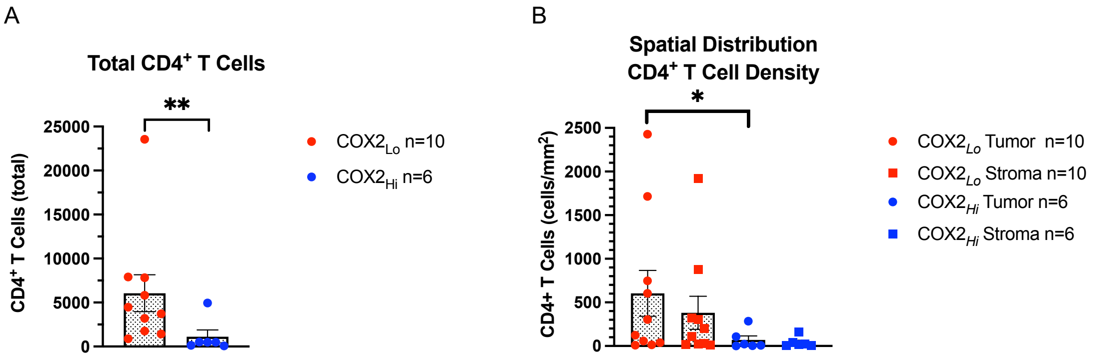

Supplemental Figure 2

Supplemental Figure 2

$CD4^+$ T cells density and spatial distribution in $COX2_{Hi}$ vs $COX2_{Lo}$ TNBC tumors. The $CD4^+$ T cell density was evaluated in A) in whole tumor images or B) in tumor and stromal regions as determined by H&E. Statistical Mann-Whitney or Kruskal-Wallis multiple comparisons test was used. ** p = 0.0075, * p = 0.0272.

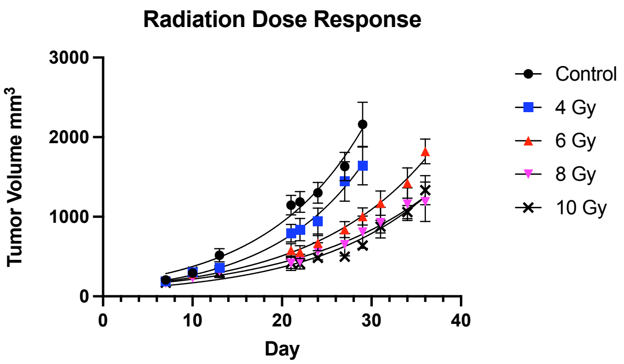

Supplemental Figure 3

Supplemental Figure 3

Radiation Dose Response Curve. Single radiation dose response was performed on 4T1 tumor bearing mice to determine an effective dose that delays tumor growth, which can be further augmented by adjuvant INDO treatment.

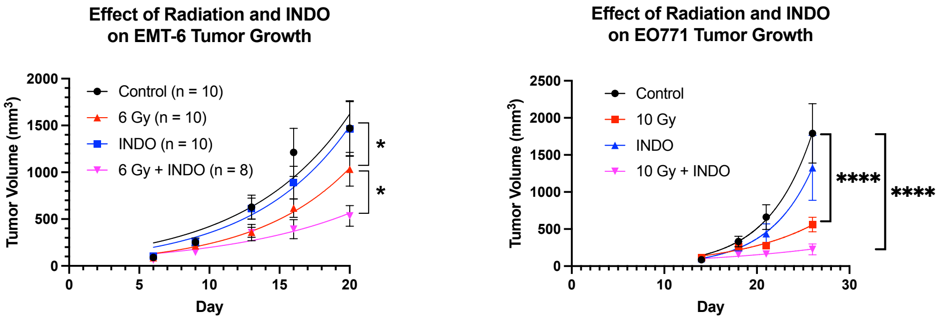

Supplemental Figure 4

Supplemental Figure 4

Indomethacin augments the radiation tumor growth delay in EMT-6 and EO771 tumor-bearing mice. A) Single doses of 6 or 10 Gy X-rays were given to EMT-6 (BALB/c) or EO771 (C57BL/6) tumor bearing mice, respectively. INDO treatment was given daily in the drinking water (30 mg/L), which began immediately following tumor irradiation and continued for the duration of the experiment. Combination treatment augmented radiation growth delay in both models.
Two-Way ANOVA + Tukey's Multiple Comparisons Test was used.

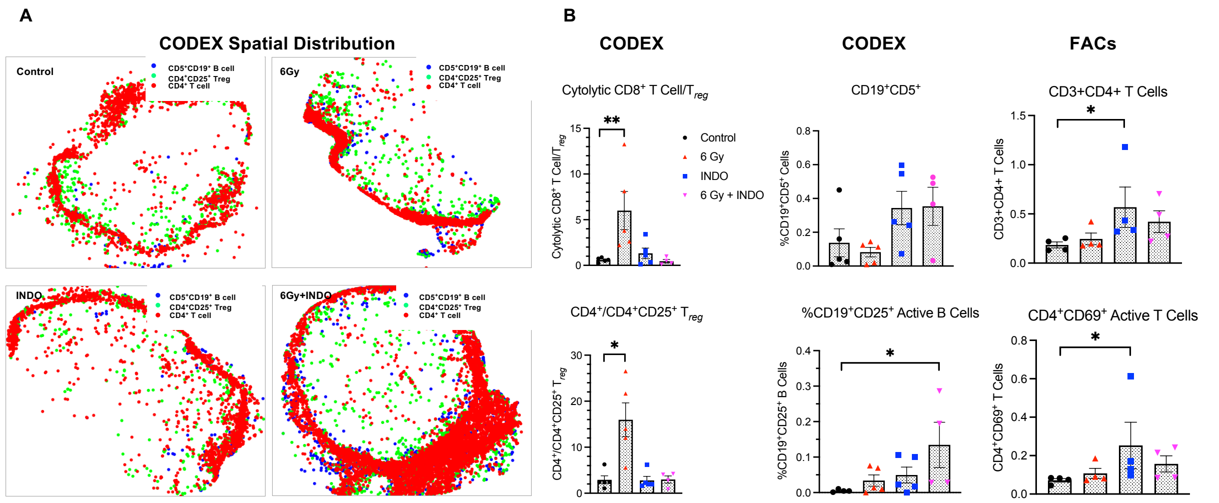

Supplemental Figure 5

Supplemental Figure 5

CODEX spatial distribution and FACs analysis of $CD4^+$ T cell and $CD19^+$ B cell phenotypes in control and treated tumors. Spatial distribution analysis represents detection $\geq$1 cell marker in a 25 µm diameter circle that defines the spatial composition of the tumor immune microenvironment. A) $CD4^+$ T cells and $CD19^+$ B cells are primarily localized at the tumor margin, with sparse infiltration of $CD4^+CD25^+$ Tregs into the tumor core. B) Quantified ratios of cytolytic $CD8^+$ and $CD4^+$ T cells-to-$CD4^+CD25^+$ T_{regs} show significantly increased ratios in 6Gy-treated tumors. CODEX analyses also showed increased $CD19^+$ B cells in INDO- and 6Gy+INDO-treated tumors while $CD19^+CD25^+$ active B cells were significantly elevated in 6Gy+INDO-treated tumors. FACs analysis shows significantly elevated $CD4^+$ T cells and $CD4^+CD69^+$ activated T cells in INDO treated tumors. * $p < 0.05$, ** $p = 0.0011$, One-way ANOVA + Dunnett's or Kruskal-Wallis was used.

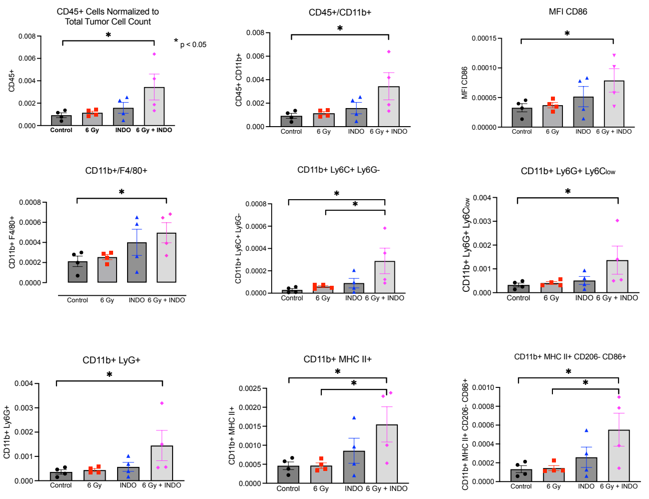

Supplemental Figure 6

Supplemental Figure 6

FACs analysis of CD45+ immune cells in control and 6 Gy +/- INDO treated 4 T1 tumors. *
*$p < 0.05$ using One-way ANOVA + Sidak's post-test.

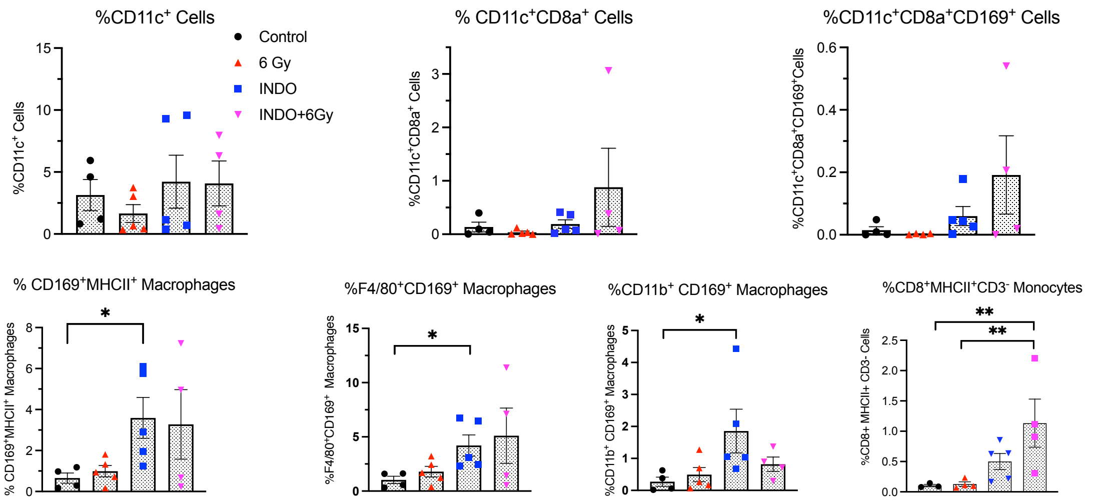

Supplemental Figure 7

Altered DC and macrophage phenotypes in INDO and 6 Gy + INDO treated tumors.

Increased trends were observed for $CD11c^{+}CD8^{+}$ DC and $CD11c^{+}CD8^{+}CD169^{+}$ macrophages known to mediate antigen presentation as well as priming and expansion of $CD8^{+}$ T cells. In addition, significant increases in $CD169^{+}$ and $CD8^{+}$ macrophages are shown in INDO- and 6Gy+INDO-treated tumors. *p < 0.05 using Dunn's or Sidak's multiple comparisons test.

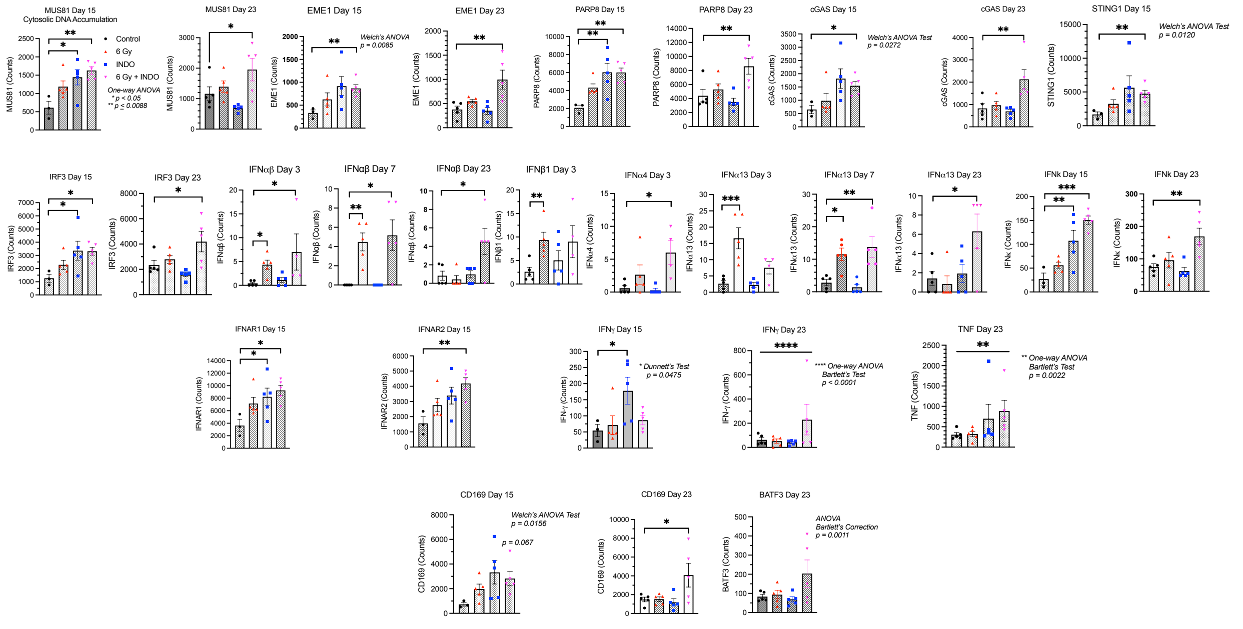

Supplemental Figure 8

Supplemental Figure 8

Increased cGAS/STING1 signaling in treated tumors over time course. Alterations of individual genes related MUS81/EME1/PARP8 DNA repair pathway leading to increased cytoplasmic tumor DNA that induces the cGAS/STING pathway and augmented type I IFN in 6Gy, INDO-, and 6Gy+INDO-treated samples. * p < 0.05, ** p < 0.01, and *** p < 0.005 using One-way ANOVA + Dunnett's post-test.

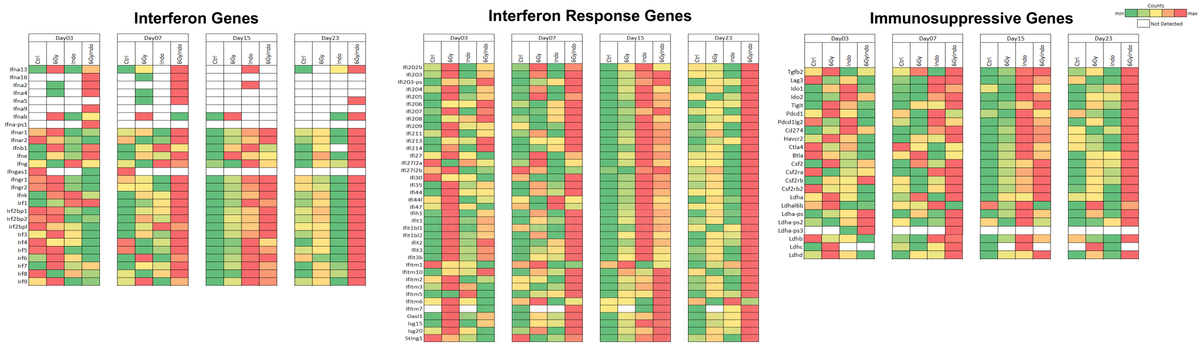

Supplemental Figure 9

Supplemental Figure 9

RNAseq gene expression analysis showing altered interferon, interferon response, and immunosuppressive gene regulation in treated tumors. The green-to-red (low-to-high) color scale indicates the number of transcript counts; white boxes indicate no transcripts were found. Prior to heat map development in Microsoft 64-bit Excel 365, transcript counts were normalized using the default "counts per million + 0.0001" method in the Partek Flow software (Build 10.0.22.0428).

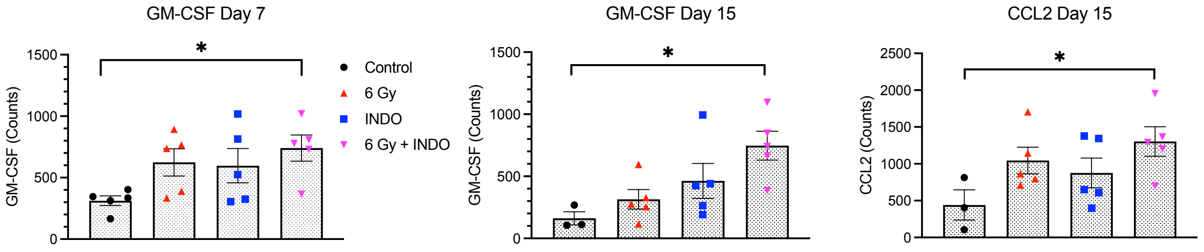

Supplemental Figure 10

Supplemental Figure 10

RNAseq gene expression analysis of GM-CSF and chemokine CCL2 in control and treated tumors at day 7 and 15. * $p < 0.05$ One-way ANOVA + Dunnettt's or Dunn's post-test were used.

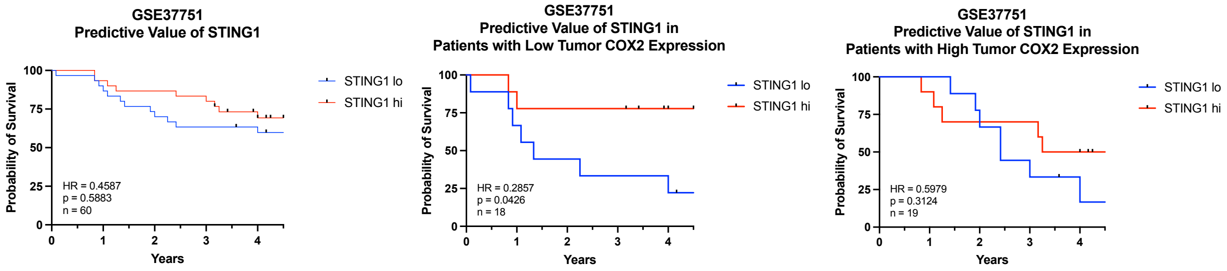

Supplemental Figure 11

Supplemental Figure 11
The probability of survival determined in GSE37751 database.
Tumor COX2 expression influences the probability of survival in TNBC patients stratified for high vs low STING1. Statistical Log-rank Mantel-Cox test was used.
